# Changes in the living arrangement and risk of stroke in Japan; does it matter who lives in the household? Who among the family matters?

**DOI:** 10.1371/journal.pone.0173860

**Published:** 2017-04-13

**Authors:** Ehab Salah Eshak, Hiroyasu Iso, Kaori Honjo, Ai Noda, Norie Sawada, Shoichiro Tsugane, on behalf of The Japan Public Health based Cohort (JPHC) study

**Affiliations:** 1 Department of Public Health and Community Medicine, Faculty of Medicine, Minia University, Minia, Egypt; 2 Public Health, Department of Social Medicine, Osaka University Graduate School of Medicine, Suita, Osaka, Japan; 3 Epidemiology and Prevention Division, Research Center for Cancer Prevention and Screening, National Cancer Center, Tokyo, Japan; Seoul National University, REPUBLIC OF KOREA

## Abstract

Previous studies have suggested associations of family composition with morbidity and mortality; however, the evidence of associations with risk of stroke is limited. We sought to examine the impact of changes in the household composition on risk of stroke and its types in Japanese population. Cox proportional hazard modelling was used to assess the risk of incident stroke and stroke types within a cohort of 77,001 Japanese men and women aged 45–74 years who experienced addition and/or loss of family members [spouse, child(ren), parent(s) and others] to their households over a five years interval (between 1990–1993 and 1995–1998). During 1,043,446 person-years of the follow-up for 35,247 men and 41,758 women, a total of 3,858 cases of incident stroke (1485 hemorrhagic and 2373 ischemic) were documented. When compared with a stable family composition, losing at least one family member was associated with 11–15% increased risk of stroke in women and men; hazard ratios (95% confidence interval) were 1.11 (1.01–1.22) and 1.15 (1.05–1.26), respectively. The increased risk was associated with the loss of a spouse, and was evident for ischemic stroke in men and hemorrhagic stroke in women. The addition of any family members to the household was not associated with risk of stroke in men, whereas the addition of a parent (s) to the household was associated with increased risk in women: 1.49 (1.09–2.28). When the loss of a spouse was accompanied by the addition of other family members to the household, the increased risk of stroke disappeared in men: 1.18 (0.85–1.63), but exacerbated in women: 1.58 (1.19–2.10). In conclusion, men who have lost family members, specifically a spouse have higher risk of ischemic stroke, and women who gained family members; specifically a parent (s) had the higher risk of hemorrhagic stroke than those with a stable family composition.

## Introduction

Despite rapid decrease in stroke mortality during the last decade in Japan, the incidence of stroke is still as high as that of Western population [1]. Epidemiological studies have shown that family composition is an important factor in determining risk of stroke [2, 3], and physical and mental health as a whole [4–9]. A number of studies have discussed the hazards and benefits of specific types of family compositions for health: the risks associated with being single [2,4], the risks associated with being a single mother [4,6], the benefits of living with a spouse [7–9], and the mixed risk/benefits of living in a multi-generational family [4,5,7].

Japanese society is currently undergoing major shifts, including rapid population aging, declining marriage and fertility rates [10]. The lives of Japanese men and women aged 45 to 74 years are dynamic; and individuals are likely to experience numerous changes in their living arrangement. Recent national statistics have indicated that there has been an increase in the proportions of persons living alone, and co-residing with aging parents [10–12] for both middle-aged women and married couples. They also indicated a decrease in the immanence of multi-generational households and of married couples living with their own [10–12].

Previous studies have examined the health impact of cause-specific major life events, such as death of a spouse [13], children [14], siblings [15] and/or parents [16]. One study has examined the potential effects of some major life events including separations from a close family member, the departure of a household member, the movement in of a new household member or a member's relocation of residence on risk of cardiac arrest [17]. Such investigations have not been yet done for risk of stroke and its types.

A recent study has shown that marital transition, defined as a change in marital status during a given time was associated with risk of stroke in Japanese men and women, and the associations were modified by accompanied changes in living arrangement [18]. However, although marital transition has two directions, that study dealt with only marital transition from married to unmarried.

No known study has examined the health impact, especially on stroke risk, of losing or gaining family members due to a variety of situations including: death, immigration, separation, divorce, or other circumstances that would create a shifting in or out of family members in a dynamic community like in Japan. Therefore, we hypothesized that these situations, categorized as experiences of loss, gain, or a mixture of both losing and gaining of family members, might have an impact on risk of stroke among Japanese men and women and we aimed to examine that through the use of data from a large nationwide cohort study, the Japan Public Health based Cohort (JPHC) study.

## Subjects and methods

### Design and population

The JPHC Study, a large population-based prospective study of 140,420 men and women aged 40–69 years, was launched in 5 Public Health Centers (PHC) for Cohort I in 1990, and 6 PHCs for Cohort II in 1993. The study design was previously described in detail [19]. Two PHC areas in metropolitan Tokyo and Osaka were excluded from the present analysis (n = 23,524) because no data on stroke incidence were available.

A self-administered questionnaire was distributed to all registered participants in cohort 1 cohort 2; (response rate: 81.6%). A follow-up survey was conducted 5 years after the first survey (response rate: 84.9%). A total of 80,964 men and women responded to both questionnaires. For this study, we set the dates of the first survey as our prebaseline and those of the second survey as our baseline to determine changes in the living arrangement between first and second surveys.

We further excluded 1,014 participants due to missing data regarding their living arrangement at either at prebaseline or baseline. In addition, 2,949 participants were also excluded due to a previous reported history of cardiovascular disease or cancer before baseline, which left a total of 35,244 men and 41,757 women aged at baseline 45–64 years for Cohort I, and 45–74 years for Cohort II. This study was approved by the Institutional Review Boards of the National Cancer Centre of Japan and Osaka University.

### Assessment of living arrangement

In the prebaseline and the baseline questionnaires, each participant answered the following question about his/her living arrangement: “Are you living with someone (alone, spouse, child(ren), parent(s), others)?”. In the Japanese culture “others” is understood to mean other family members, i.e. siblings, grandparents, uncle, aunts, cousins, in-laws etc. Based on the answers to this question from the prebaseline and the baseline surveys, participants were classified into the following groups: “No change in the living arrangement”, “Losing one or more category of family members”, “Gaining one or more category of family members”, and “Mixed losing and gaining”.

When participants had reported only losing family members (one or more members reported as part of their household initially, were reported as no longer present), we classified them into the following subgroups: “Losing a spouse”, “Losing a child(ren)”, “Losing a parent(s)”, and “Losing others”. Those who had experienced only gaining family members were classified into the following subgroups: “Gaining a spouse”, “Gaining a child(ren)”, “Gaining a parent(s)”, and “Gaining others”. Participants who experienced a combination of losing some family members, while gaining new members were classified twice. First they were classified in accordance to the category of family member they had “lost”: “Losing a spouse and gaining any”, “Losing a child(ren) and gaining any”, “Losing a parent(s) and gaining any”, and “Losing others and gaining any”. They were then also classified according to the “gained” category: “Gaining a spouse and losing any”, “Gaining a child(ren) and losing any”, “Gaining a parent(s) and losing any”, and “Gaining others and losing any”.

### Endpoint assessment

A total of 78 major hospitals with the capability of treating patients from stroke were registered within the administrative districts of the JPHC cohort. Physicians, unaware of the patients’ lifestyle data, reviewed the medical records at each hospital. Incidences of stroke and its types were confirmed if the criteria of the National Survey of Stroke [20] were met, specifically, the presence of focal neurological deficits of sudden or rapid onset lasting for at least 24 hours or until death. Definite diagnosis for hemorrhagic and ischemic strokes was established on the basis of data collected from computed tomography scans, magnetic resonance images, or autopsy.

### Statistical analyses

Age-adjusted mean values and proportions of population characteristics at the baseline were presented according to the types of changes in the living arrangement. Person-years of follow-up were calculated from the baseline, January 1, 1995 in Cohort I, and January 1, 1998 in Cohort II until one of four end points first occurred: incidence of the first stroke event, death, relocation from the study area, or the end of the follow-up (December 31, 2009 in Cohort I and December 31, 2012 in Cohort II). For persons who could not be located for a follow-up survey, the last confirmed date of their presence in the study area was used as their exit date.

Sex-specific hazard ratios (HR) with 95% confidence intervals (CI) for risk of incident stroke and its types were calculated by using the Cox proportional hazard modeling in the three groups of changes in the living arrangement: “Losing ≥1 category of family members”, “Gaining ≥1 category of family members”, and “Mixed losing and gaining”, and compared with the risk in the “No change in the living arrangement” group, which was used as a reference. Further detailed analyses were conducted in each group according to the category of the loss or gain of family members, and the risk in each of these subgroups was also compared to that in the “No change in the living arrangement” group.

We adjusted for age (continuous) and residential area in the first model, and further adjusted for other hypothesized confounding factors including histories of hypertension, diabetes and use of cholesterol-lowering drugs (yes or no), occupation (white-collar job, blue collar job and unemployed) and the number of cohabitants at baseline in the second model. Further adjustment for possible mediators including BMI (quintiles); physical activity (quintiles of METs units); smoking status (never, ex-smoker, current smoker of 1–19, 20–29, or ≥30 cigarettes per day); ethanol intake (nondrinkers, former drinkers, and weekly ethanol intake of <150 g/wk, 150- <300 g/wk, 300-<450 g/wk, or ≥450 g/wk); perceived psychological stress (low, moderate, and high) and life enjoyment (yes or no), were done in a third mode. These characteristics might be considered consequences of changes in living arrangement because such changes occurred before the baseline.

Varying levels of loss were reported within the losing subgroups, as well as varying levels of gain within the gaining subgroups (for example, a participant who had lost a spouse may have also lost a child(ren), parent(s), and/or others, while a participant who gained a spouse could also gained a child(ren), parent(s), and/or others and etc.). In order to account for loss independent of gaining family members, in the detailed analyses of subgroups for those with losing experience, we further adjusted in the second model for losing any other categories of family members other than the targeted one (yes or no) and, likewise, we further adjusted for gaining any other categories of family members (yes or no) in the gaining subgroups. In addition sensitivity analyses were conducted among those who had experienced one change only (eg, only losing a spouse, only gaining parents).

For participants who reported mixed losing and gaining of family members we further adjusted in the second model for the net result difference in the number of cohabitants at baseline compared with the number of cohabitants at prebaseline survey (number of cohabitants at prebaseline = number of cohabitants at baseline, prebaseline cohabitants < baseline cohabitants, or prebaseline cohabitants> baseline cohabitants). The analysis was conducted with SAS version 9.4 (SAS Institute Inc, Cary, NC). All P values are 2-sided, and P<0.05 was regarded as statistically significance.

## Results

The characteristics of study participants who experienced changes in their family household composition when compared to those with no change are shown in **Table 1**. Both men and women who experienced changes of their living arrangement were more likely to smoke, to be hypertensive and to have more stress and less life enjoyment.

**Table 1:** Participants’ Characteristics at Baseline According to Changes in the Living Arrangement.

	Changes in Living Arrangement			
	No Change	Losing ≥ 1 Family Member	Gaining ≥ 1 Family Member	Mixed Type Losing and Gaining
Men at risk, *n*	19491	9992	3443	2318
Age, year	56 (8)	57 (7)	58 (8)	56 (7)
Body mass index, kg/m^2^	23.6 (3.1)	23.6 (3.2)	23.5 (3.1)	23.5 (3.2)
Current smokers, %	49	52	50	52
Ethanol intake, g/week	303 (259)	323 (293)	306 (252)	325 (291)
Physical activity, METs unites/week	34 (7)	33 (7)	35 (7)	34 (7)
History of hypertension, %	19	21	21	19
History of diabetes, %	7	7	7	7
Economically active, %	90	92	91	91
Perceived high mental stress,%	19.4	20.6	20.9	20.7
Life enjoyment	14.3	12.6	11.9	13.6
Number of cohabitants at prebaseline, *n*	1.9 (0.78)	2.3 (0.67)	1.4 (0.70)	2.0 (0.81)
Number of cohabitants at baseline, *n*	1.9 (0.78)	1.1 (0.70)	2.5 (0.69)	1.8 (0.81)
Losing a spouse, %	0	26.9	0	24.3
Losing a child(ren), %	0	54.1	0	27.9
Losing a parent(s), %	0	32.7	0	49.9
Losing others, %	0	9.7	0	17.0
Gaining a spouse, %	0	0	25.3	26.6
Gaining a child(ren), %	0	0	39.4	17.3
Gaining a parent(s), %	0	0	16.0	17.6
Gaining “others”, %	0	0	32.5	46.9
Women at risk, *n*	21722	14465	4280	3290
Age, year	57 (8)	57 (8)	58 (7)	57 (7)
Body mass index, kg/m^2^	23.6 (3.5)	23.6 (3.6)	23.7 (3.6)	23.5 (3.6)
Current smokers, %	11	14	11	12
Ethanol intake, g/week	88 (169)	96 (147)	82 (139)	69 (108)
Physical activity, METs unites/week	33 (6)	33 (6)	34 (6)	33 (5)
History of hypertension, %	20	22	22	21
History of diabetes, %	4	4	3	3
Economically active, %	51	59	54	55
Perceived high mental stress,%	16.3	19.5	20.3	18.7
Life enjoyment	18.1	16.7	14.9	14.2
Number of cohabitants at prebaseline, *n*	1.6 (0.81)	2.2 (0.70)	1.3 (0.68)	1.9 (0.78)
Number of cohabitants at baseline, *n*	1.6 (0.81)	1.0 (0.69)	2.4 (0.70)	1.8 (0.77)
Losing a spouse, %	0	35.1	0	32.8
Losing a child(ren), %	0	48.2	0	27.7
Losing a parent(s), %	0	25.2	0	40.4
Losing others, %	0	12.0	0	16.4
Gaining a spouse, %	0	0	26.8	25.4
Gaining a child(ren), %	0	0	32.9	19.4
Gaining a parent(s), %	0	0	14.3	18.1
Gaining “others”, %	0	0	37.1	44.5

**Table 2**shows the hazard ratios (HRs) of stroke and its types according to groups of changes in the family household composition. During the 1,043,446 person-years of the follow-up (median follow-up period = 13.6 years) for 35,247 men and 41,758 women, a total of 3,858 cases of newly diagnosed stroke (1485 hemorrhagic and 2373 ischemic) were documented. When compared with no change in the household composition, men and women who have lost ≥1 category of family members have higher risk of total stroke; the multivariable HRs (95% CI) were 1.15 (1.05–1.26) and 1.11 (1.01–1.22), respectively (model 2). Among women only, gaining ≥1 category of family members and mixed losing and gaining of family members were associated with higher risk of stroke; HRs (95% CI) were 1.16 (1.02–1.37) and 1.22 (1.02–1.46), respectively (model 2). These associations were evident for ischemic stroke in men and hemorrhagic stroke in women, and remained unchanged after further adjustment for possible mediating factors (model 3).

**Table 2:** Adjusted Hazard Ratios for Incident Stroke According to Changes in the Living Arrangement Within 5 Years in Japanese Men and Women.

	Changes in Living Arrangement			
	No Change	Losing ≥ 1 Family Member(s)	Gaining ≥ 1 Family Member(s)	Mixed Type of Losing and Gaining Family Members
**Men** at risk, *n*	19491	9992	3443	2318
Person-year	256989	130193	45384	30676
**Total Stroke**				
Cases, n	1160	657	226	148
Model 1 ^a^	1.00	1.17 (1.07–1.28)	1.03 (0.89–1.19)	1.08 (0.91–1.29)
Model 2 ^b^	1.00	1.15 (1.05–1.26)	1.05 (0.91–1.22)	1.09 (0.92–1.29)
Model 3 ^c^	1.00	1.14 (1.02–1.23)	1.03 (0.88–1.20)	1.05 (0.89–1.25)
**Hemorrhagic stroke**				
Cases, *n*	425	205	71	46
Model 1 ^a^	1.00	0.92 (0.78–1.09)	0.90 (0.70–1.16)	0.91 (0.67–1.23)
Model 2 ^b^	1.00	0.93 (0.70–1.00)	0.98 (0.75–1.28)	0.90 (0.67–1.22)
Model 3 ^c^	1.00	0.81 (0.67–0.97)	0.96 (0.73–1.24)	0.88 (0.65–1.19)
**Ischemic stroke**				
Cases, *n*	735	452	155	102
Model 1 ^a^	1.00	1.16 (1.03–1.30)	1.10 (0.93–1.31)	1.19 (0.97–1.47)
Model 2 ^b^	1.00	1.16 (1.02–1.31)	1.10 (0.91–1.32)	1.20 (0.97–1.48)
Model 3 ^c^	1.00	1.12 (0.99–1.27)	1.07 (0.89–1.28)	1.15 (0.94–1.42)
**Women** at risk, n	21722	12465	4280	3290
Person-year	301153	173231	59909	45911
**Total Stroke**				
Cases, n	829	498	193	147
Model 1 ^a^	1.00	1.10 (1.00–1.23)	1.16 (0.99–1.35)	1.21 (1.02–1.44)
Model 2 ^b^	1.00	1.11 (1.01–1.22)	1.16 (1.02–1.37)	1.22 (1.02–1.46)
Model 3 ^c^	1.00	1.09 (0.98–1.18)	1.16 (1.00–1.36)	1.21 (1.02–1.46)
**Hemorrhagic stroke**				
Cases, *n*	350	241	81	66
Model 1 ^a^	1.00	1.18 (0.99–1.40)	1.28 (1.02–1.73)	1.27 (0.98–1.65)
Model 2 ^b^	1.00	1.19 (1.00–1.42)	1.29 (1.01–1.74)	1.29 (0.99–1.67)
Model 3 ^c^	1.00	1.18 (0.99–1.41)	1.27 (1.00–1.70)	1.27 (0.98–1.66)
**Ischemic stroke**				
Cases, *n*	479	257	112	81
Model 1 ^a^	1.00	0.96 (0.82–1.11)	1.17 (0.90–1.44)	1.18 (0.90–1.49)
Model 2 ^b^	1.00	1.00 (0.85–1.17)	1.13 (0.88–1.41)	1.17 (0.90–1.49)
Model 3 ^c^	1.00	0.99 (0.84–1.16)	1.12 (0.87–1.39)	1.17 (0.90–1.49)

^a^ Model 1 Adjusted for age and residential area.

^b^ Model 2 Adjusted further for histories of hypertension, diabetes and use of cholesterol-lowering drugs, job status and number of cohabitants at baseline time.

^c^ Model 3 Adjusted further for body mass index, physical activity, smoking status, ethanol intake, perceived psychological stress, life enjoyment.

**Tables 3 and 4**show the HRs for stroke risk within subgroups of losing and gaining family members. Losing a spouse was associated with higher risk of total and ischemic strokes in men and tended to increase the risk of total and hemorrhagic strokes in women. Due to overlap within the subgroups for these analyses, adjustments were made for the loss of other family members besides the loss of spouse; the multivariable HRs (95% CI) for total stroke were 1.17 (1.09–1.39) for men and 1.11 (0.98–1.33) for women (model 2). Moreover, when the analysis was restricted to only those who experienced loss of only a spouse (excluding members lost spouse and other family members) the results did not materially change; HRs (95% CI) in men and women were 1.15 (1.04–1.39) and 1.11 (0.99–1.38), respectively. Losing family members other than spouse was not associated with risk of stroke in either gender.

**Table 3:** Adjusted Hazard Ratios for Incident Stroke According to Losing Specific Categories of Household Members Within 5 Years in Japanese Men and Women.

	Losing a Household Member				
	No Change	Losing a Spouse	Losing a Child(ren)	Losing a Parent(s)	Losing Others
**Men** at risk, *n*	19491	2686	5409	3265	968
Person-year	256989	34250	70690	43416	12333
Total Stroke					
Cases, *n*	1160	201	346	202	72
Model 1 ^a^	1.00	1.17 (1.08–1.36)	1.03 (0.92–1.16)	1.03 (0.88–1.18)	1.03 (0.82–1.30)
Model 2 ^b^	1.00	1.17 (1.09–1.39)	1.01 (0.88–1.16)	1.00 (0.85–1.17)	1.01 (0.79–1.30)
Model 3 ^c^	1.00	1.12 (1.02–1.35)	0.99 (0.86–1.15)	0.99 (0.84–1.17)	0.98 (0.77–1.26)
Hemorrhagic Stroke					
Cases, *n*	1160	63	112	52	23
Model 1 ^a^	1.00	1.12 (0.86–1.45)	0.96 (0.78–1.18)	0.72 (0.54–0.96)	1.03 (0.68–1.57)
Model 2 ^b^	1.00	0.95 (0.69–1.32)	0.86 (0.68–1.10)	0.67 (0.49–0.92)	1.00 (0.65–1.57)
Model 3 ^c^	1.00	0.89 (0.64–1.23)	0.86 (0.67–1.10)	0.67 (0.49–0.92)	0.96 (0.62–1.49)
Ischemic Stroke					
Cases, *n*	1160	138	234	150	49
Model 1 ^a^	1.00	1.20 (1.01–1.44)	1.07 (0.93–1.44)	1.20 (1.01–1.43)	1.02 (0.77–1.36)
Model 2 ^b^	1.00	1.26 (1.01–1.58)	1.10 (0.92–1.31)	1.20 (0.99–1.45)	1.01 (0.74–1.36)
Model 3 ^c^	1.00	1.24 (0.99–1.54)	1.08 (0.90–1.28)	1.18 (0.97–1.44)	0.99 (0.73–1.34)
**Women** at risk, *n*	21722	4373	6006	3141	1492
Person-year	301153	60078	84084	44081	20537
Cases, *n*	829	199	217	106	82
Model 1 ^a^	1.00	1.13 (1.00–1.35)	1.01 (0.88–1.17)	0.97 (0.79–1.18)	1.18 (0.94–1.47)
Model 2 ^b^	1.00	1.11 (0.98–1.33)	1.07 (0.90–1.27)	0.97 (0.78–1.21)	1.16 (0.90–1.50)
Model 3 ^c^	1.00	1.10 (0.98–1.32)	1.07 (0.90–1.32)	0.99 (0.79–1.23)	1.17 (0.89–1.48)
Hemorrhagic Stroke					
Cases, *n*	829	92	110	48	40
Model 1 ^a^	1.00	1.19 (0.99–1.55)	1.13 (0.91–1.39)	0.93 (0.69–1.25)	1.27 (0.90–1.90)
Model 2 ^b^	1.00	1.23 (1.00–1.59)	1.17 (0.92–1.50)	0.97 (0.70–1.34)	1.35 (0.92–2.06)
Model 3 ^c^	1.00	1.20 (0.99–1.57)	1.20 (0.93–1.53)	0.98 (0.71–1.35)	1.32 (0.90–2.02)
Ischemic Stroke					
Cases, *n*	829	107	107	58	42
Model 1 ^a^	1.00	1.01 (0.82–1.24)	0.93 (0.75–1.14)	1.01 (0.77–1.32)	1.04 (0.76–1.42)
Model 2 ^b^	1.00	1.05 (0.82–1.34)	0.98 (0.77–1.25)	0.98 (0.73–1.32)	1.00 (0.71–1.40)
Model 3 ^c^	1.00	1.03 (0.81–1.32)	0.98 (0.76–1.24)	1.00 (0.74–1.34)	1.01 (0.72–1.42)

^a^ Model 1 Adjusted for age and residential area.

^b^ Model 2 Adjusted further for histories of hypertension, diabetes and use of cholesterol-lowering drugs, job status, number of cohabitants at baseline and for losing any other members in the family.

^c^ Model 3 As model 2 and adjusted further for body mass index, physical activity, smoking status, ethanol intake, perceived psychological stress and life enjoyment.

**Table 4:** Adjusted Hazard Ratios for Incident Stroke According to Gaining Specific Categories of Household Members Within 5 Years in Japanese Men and Women.

	Gaining a Household Member				
	No Change	Gaining a Spouse	Gaining a Child(ren)	Gaining a Parent(s)	Gaining Others
**Men** at risk, *n*	19491	870	1357	551	1120
Person-year	256989	11644	17812	7621	14394
Cases, *n*	1160	59	92	24	83
Model 1 ^a^	1.00	1.33 (1.02–1.72)	0.98 (0.79–1.21)	0.82 (0.54–1.22)	1.01 (0.81–1.26)
Model 2 ^b^	1.00	1.26 (0.94–1.64)	0.97 (0.77–1.23)	0.82 (0.54–1.25)	1.02 (0.79–1.32)
Model 3 ^c^	1.00	1.20 (0.86–1.46)	0.97 (0.76–1.22)	0.81 (0.53–1.23)	1.00 (0.77–1.29)
Hemorrhagic Stroke					
Cases, *n*	1160	19	28	9	26
Model 1 ^a^	1.00	1.11 (0.70–1.76)	0.87 (0.59–1.28)	0.80 (0.41–1.56)	0.97 (0.65–1.44)
Model 2 ^b^	1.00	1.09 (0.66–1.80)	0.88 (0.57–1.35)	0.86 (0.43–1.71)	1.13 (0.72–1.78)
Model 3 ^c^	1.00	1.06 (0.64–1.74)	0.87 (0.57–1.34)	0.84 (0.42–1.69)	1.11 (0.70–1.75)
Ischemic Stroke					
Cases, *n*	1160	40	64	15	57
Model 1 ^a^	1.00	1.36 (0.93–2.02)	1.03 (0.80–1.34)	0.82 (0.49–1.38)	1.03 (0.79–1.35)
Model 2 ^b^	1.00	1.36 (0.90–2.06)	1.02 (0.77–1.35)	0.80 (0.47–1.37)	0.95 (0.69–1.31)
Model 3 ^c^	1.00	1.32 (0.91–2.02)	1.01 (0.76–1.34)	0.78 (0.46–1.34)	0.93 (0.68–1.27)
**Women** at risk, *n*	21722	1145	1408	610	1587
Person-year	301153	16048	19646	8674	22134
Cases, *n*	829	49	61	28	82
Model 1 ^a^	1.00	1.28 (0.96–1.70)	1.01 (0.78–1.31)	1.48 (1.11–2.15)	1.10 (0.87–1.37)
Model 2 ^b^	1.00	1.27 (0.93–1.72)	1.00 (0.76–1.33)	1.49 (1.09–2.28)	1.03 (0.79–1.35)
Model 3 ^c^	1.00	1.13 (0.85–1.62)	0.98 (0.74–1.30)	1.42 (1.07–2.14)	1.01 (0.77–1.33)
Hemorrhagic Stroke					
Cases, *n*	829	20	27	17	31
Model 1 ^a^	1.00	1.13 (0.73–1.77)	1.07 (0.73–1.59)	1.94 (1.20–3.16)	1.03 (0.72–1.49)
Model 2 ^b^	1.00	1.07 (0.66–1.74)	1.05 (0.68–1.61)	1.89 (1.15–3.26)	1.05 (0.68–1.63)
Model 3 ^c^	1.00	1.06 (0.65–1.72)	1.03 (0.67–1.33)	1.86 (1.12–2.22)	1.00 (0.65–1.56)
Ischemic Stroke					
Cases, *n*	829	29	34	11	51
Model 1 ^a^	1.00	1.32 (0.91–2.07)	0.96 (0.68–1.36)	1.08 (0.59–1.96)	1.15 (0.86–1.53)
Model 2 ^b^	1.00	1.35 (0.91–2.16)	0.98 (0.68–1.41)	1.03 (0.55–1.94)	1.02 (0.73–1.43)
Model 3 ^c^	1.00	1.34 (0.87–2.14)	0.95 (0.65–1.37)	1.05 (0.56–1.98)	1.02 (0.73–1.44)

^a^ Model 1 Adjusted for age and residential area.

^b^ Model 2 Adjusted further for histories of hypertension, diabetes and use of cholesterol-lowering drugs, job status, number of cohabitants at baseline and for gaining any other members in the family.

^c^ Model 3 As model 2 and adjusted further for body mass index, physical activity, smoking status, ethanol intake, perceived psychological stress and life enjoyment.

Gaining family members was not associated with risk of stroke in men; whereas, gaining a parent(s) was associated with increased risk of total and hemorrhagic strokes among women; HRs (95%CI) were 1.49 (1.09–2.28) and 1.89 (1.15–3.26), respectively. Analysis restricted to women who experienced only gaining a parent(s) with no other change in family composition revealed similar associations; HRs (95%CI) were 1.39 (1.07–2.14) and 1.66 (1.13–3.11), respectively.

There were no association of any combination of mixed losing and gaining of family members with risk of stroke in men as shown in **Tables 5 and 6**. However, in women, the loss of a spouse combined with the gain of any other category(ies) of family members, as well as the gain of a child(ren) or a parent(s) combined with the loss of any other category(ies) of family members, were associated with a higher risk of stroke, which was evident for total and hemorrhagic strokes in women who gained a child(ren) or a parent(s) while lost a spouse **(S1 Table)**.

**Table 5:** Adjusted Hazard Ratios for Incident Stroke in the Mixed Losing and Gaining Group According to the Lost Category of Household Members Within 5 Years in Japanese Men and Women.

	Mixed Change According to the Lost Household Member				
	No Change	Losing a Spouse-Gaining Any	Losing a Child(ren)-Gaining Any	Losing a Parent(s)-Gaining Any	Losing Others-Gaining Any
Men at risk, *n*	19491	563	647	1156	393
Person-year	256989	7117	8572	15599	5285
Cases, *n*	1160	48	43	63	20
Model 1 ^a^	1.00	1.28 (0.95–1.72)	1.10 (0.80–1.51)	0.93 (0.72–1.21)	0.87 (0.56–1.36)
Model 2 ^b^	1.00	1.18 (0.85–1.63)	1.09 (0.79–1.50)	0.85 (0.62–1.16)	0.84 (0.52–1.35)
Model 3 ^c^	1.00	1.19 (0.86–1.65)	0.97 (0.70–1.34)	0.84 (0.49–1.26)	0.78 (0.49–1.26)
Hemorrhagic Stroke					
Cases, *n*	1160	14	11	25	5
Model 1 ^a^	1.00	1.16 (0.67–2.00)	0.76 (0.41–1.42)	1.05 (0.70–1.58)	0.60 (0.25–1.46)
Model 2 ^b^	1.00	1.15 (0.63–2.11)	0.77 (0.41–1.43)	1.03 (0.61–1.76)	0.63 (0.25–1.59)
Model 3 ^c^	1.00	1.11 (0.61–2.03)	0.76 (0.41–1.42)	1.03 (0.61–1.75)	0.62 (0.24–1.59)
Ischemic Stroke					
Cases, *n*	1160	34	32	38	15
Model 1 ^a^	1.00	1.34 (0.94–1.91)	1.30 (0.90–1.89)	0.87 (0.63–1.21)	1.03 (0.62–1.72)
Model 2 ^b^	1.00	1.19 (0.81–1.76)	1.28 (0.88–1.86)	0.77 (0.53–1.14)	0.96 (0.55–1.67)
Model 3 ^c^	1.00	1.14 (0.77–1.69)	1.26 (0.87–1.83)	0.75 (0.51–1.13)	0.95 (0.55–1.65)
Women at risk, *n*	21722	1079	912	1330	539
Person-year	301153	14718	12751	18868	7542
Cases, *n*	829	74	43	43	17
Model 1 ^a^	1.00	1.54 (1.21–1.96)	1.32 (0.97–1.82)	0.87 (0.64–1.18)	0.85 (0.52–1.37)
Model 2 ^b^	1.00	1.58 (1.19–2.10)	1.32 (0.96–1.82)	0.89 (0.64–1.24)	0.85 (0.52–1.40)
Model 3 ^c^	1.00	1.60 (1.21–2.13)	1.22 (0.88–1.68)	0.92 (0.66–1.28)	0.78 (0.74–1.28)
Hemorrhagic Stroke					
Cases, *n*	829	36	20	22	6
Model 1 ^a^	1.00	1.86 (1.31–2.64)	1.32 (0.83–2.09)	0.96 (0.62–1.49)	0.67 (0.30–1.50)
Model 2 ^b^	1.00	2.05 (1.37–3.07)	1.33 (0.83–2.13)	1.05 (0.66–1.66)	0.74 (0.32–1.68)
Model 3 ^c^	1.00	2.05 (1.37–3.06)	1.35 (0.84–2.19)	1.07 (0.67–1.70)	0.74 (0.32–1.68)
Ischemic Stroke					
Cases, *n*	829	38	23	21	11
Model 1 ^a^	1.00	1.32 (0.95–1.85)	1.34 (0.88–2.06)	0.79 (0.51–1.23)	0.98 (0.54–1.79)
Model 2 ^b^	1.00	1.25 (0.83–1.87)	1.27 (0.82–1.98)	0.76 (0.47–1.22)	0.89 (0.47–1.69)
Model 3 ^c^	1.00	1.24 (0.83–1.86)	1.26 (0.81–1.96)	0.78 (0.48–1.27)	0.90 (0.48–1.70)

^a^ Model 1 Adjusted for age and residential area.

^b^ Model 2 Adjusted further for histories of hypertension, diabetes and use of cholesterol-lowering drugs, job status and the net total categories of family member at time of prebaseline and baseline surveys.

^c^ Model 3 Adjusted further for body mass index, physical activity, smoking status, ethanol intake, perceived psychological stress and life enjoyment.

**Table 6:** Adjusted Hazard Ratios for Incident Stroke in the Mixed Losing and Gaining Group According to the Gained Category of Household Members Within 5 Years in Japanese Men and Women.

	Mixed Change According to the Gained Household Member				
	No Change	Gaining a Spouse-Losing Any	Gaining a Child(ren)-Losing Any	Gaining a Parent(s)-Losing Any	Gaining Others-Losing Any
Men at risk, *n*	19491	616	402	409	1086
Person-year	256989	8172	5230	5540	14339
Cases, *n*	1160	43	28	18	69
Model 1 ^a^	1.00	1.30 (0.96–1.77)	0.99 (0.68–1.44)	0.80 (0.50–1.27)	1.08 (0.84–1.37)
Model 2 ^b^	1.00	1.28 (0.89–1.86)	0.95 (0.63–1.42)	0.75 (0.45–1.25)	1.04 (0.75–1.25)
Model 3 ^c^	1.00	1.09 (0.75–1.59)	0.93 (0.62–1.40)	0.70 (0.42–1.16)	1.02 (0.73–1.41)
Hemorrhagic Stroke					
Cases, *n*	1160	12	7	6	23
Model 1 ^a^	1.00	0.99 (0.55–1.74)	0.75 (0.35–1.59)	0.72 (0.32–1.62)	0.98 (0.65–1.49)
Model 2 ^b^	1.00	0.89 (0.44–1.82)	0.68 (0.30–1.55)	0.64 (0.26–1.58)	0.87 (0.47–1.61)
Model 3 ^c^	1.00	0.84 (0.41–1.72)	0.68 (0.30–1.55)	0.63 (0.26–1.57)	0.88 (0.48–1.64)
Ischemic Stroke					
Cases, *n*	1160	31	21	12	46
Model 1 ^a^	1.00	1.37 (0.95–2.16)	1.10 (0.71–1.71)	0.84 (0.48–1.49)	1.13 (0.84–1.52)
Model 2 ^b^	1.00	1.31 (0.90–2.37)	1.07 (0.67–1.71)	0.82 (0.44–1.50)	1.13 (0.76–1.67)
Model 3 ^c^	1.00	1.23 (0.78–2.28)	1.01 (0.63–1.62)	0.81 (0.44–1.49)	1.09 (0.74–1.60)
Women at risk, *n*	21722	837	639	595	1465
Person-year	301153	11766	8804	8415	20339
Cases, *n*	829	29	40	21	70
Model 1 ^a^	1.00	0.96 (0.66–1.39)	1.31 (1.15–1.80)	1.20 (0.98–1.68)	1.29 (0.91–1.65)
Model 2 ^b^	1.00	0.98 (0.65–1.46)	1.35 (1.15–1.93)	1.22 (1.01–1.72)	1.25 (0.90–1.73)
Model 3 ^c^	1.00	0.85 (0.57–1.28)	1.36 (1.16–1.94)	1.20 (1.03–1.79)	1.35 (0.90–1.73)
Hemorrhagic Stroke					
Cases, *n*	829	11	20	10	31
Model 1 ^a^	1.00	0.80 (0.44–1.46)	1.65 (1.15–2.59)	1.22 (0.99–2.11)	1.30 (0.90–1.88)
Model 2 ^b^	1.00	0.87 (0.46–1.64)	1.80 (1.20–2.95)	1.29 (1.02–2.39)	1.35 (0.91–2.23)
Model 3 ^c^	1.00	0.87 (0.46–1.64)	1.84 (1.21–3.03)	1.35 (1.05–2.43)	1.37 (0.90–2.28)
Ischemic Stroke					
Cases, *n*	829	18	20	11	39
Model 1 ^a^	1.00	1.10 (0.69–1.76)	1.08 (0.69–1.70)	1.05 (0.58–1.91)	1.29 (0.90–1.79)
Model 2 ^b^	1.00	1.04 (0.61–1.77)	1.06 (0.64–1.76)	1.01 (0.53–1.93)	1.27 (0.83–1.93)
Model 3 ^c^	1.00	1.06 (0.62–1.81)	1.07 (0.64–1.77)	1.00 (0.52–1.91)	1.28 (0.84–1.96)

^a^ Model 1 Adjusted for age and residential area.

^b^ Model 2 Adjusted further for histories of hypertension, diabetes and use of cholesterol-lowering drugs, job status and the net total categories of family members at time of prebaseline and baseline surveys.

^c^ Model 3 Adjusted further for body mass index, physical activity, smoking status, ethanol intake, perceived psychological stress and life enjoyment.

## Discussion

This study of a large, prospective, population-based Japanese cohort has identified gender differences in stroke risk associated with changes in household family composition. The loss of ≥1 family members was associated with higher risk of total and ischemic strokes in men and total and hemorrhagic strokes in women; the indicated risk was associated with the loss of a spouse. When the loss of a spouse in either men or women, was accompanied by gaining of new family members, the aforementioned elevated risk of stroke in men disappeared, while the risk observed in women increased. The gain of ≥1 family member was associated with higher risk of total and hemorrhagic strokes in women only, and the indicated risk was associated with the gain of a parent(s). Moreover, mixed losing and gaining of different family members was associated with higher risk of total and hemorrhagic strokes in women only, and the indicated risk was associated with the loss of spouse accompanied by gaining a child(ren) or a parent(s). The category of “losing a family member” in our data should not be equated with that member’s death, as it was likely due to various changes in circumstances (divorce, separation, other situations that caused a shifting out, and shifting in of the household).

The finding that losing companionship was associated with increased risk of stroke in men and women of the current study is consistent with other Japanese and Western studies which showed increased risks of morbidity and mortality after the death of a family member [13–16], divorce [18, 21], separations from a close family member, departure of a household member, movement in of a new household member or a member's relocation of residence [17,18].

The indicated increased risk of stroke upon losing family members in our study was associated with the loss of a spouse for both men and women, which was similar to previous studies in Japan [18] and Western countries [21–24]. These findings might be attributed to the psychological stress that follows such major life changing events stemming from the loss of financial stability [22], and reduced social support and social networks [9,23,24]. Perceived mental stress was positively associated with risk of stroke in Japanese women; however the association was generally weaker in men [25]. This may be mediated by short-or long-term pathophysiological changes involving the sympathetic nervous system, the hypothalamic- pituitary- adrenal axis and immune system [26]. Other hypothesized mechanisms underlying the association between losing companionship and stroke risk include adopting unhealthy profiles of health behavior [27–29], and exacerbating of preexisting health conditions [2,3,9,30]; which were evident from the baseline characteristics of our studied participants; more smokers and drinkers, higher prevalence of hypertensions, more mental stress and less life enjoyment in participants who experienced loss of ≥1 family members. Marital dissolution was associated with higher risk of ischemic and hemorrhagic strokes in Japanese and Swedish men, but only with risk of hemorrhagic stroke in Japanese women and ischemic stroke in Swedish women [18, 21]. The exact mechanisms by which changes in the living arrangement were associated with ischemic stroke in Japanese men, while associated with hemorrhagic stroke in women are not clear.

Gaining family members in our study was associated with increased risk of stroke in women but not men; the indicated risk was associated with the gain of parents. The pattern of care giving in Japan is highly gendered [7,31]; the Japanese culture places men as the main source of financial security and women as the sole caretaker of children, husband, parents and parents-in-lows [7,31, 32]. It was reported that more than 85% of those who care for elderly parents were women; meanwhile, more than 80% of Japanese wives were responsible for the majority of household tasks [7, 31]. A Japanese study has shown that the probability for institutionalization of disabled older people was significantly lower in parents living with daughters when compared with parents living with sons, regardless of the daughter’s and the son’s marital status; the odds ratio (95%CI) for institutionalization was 0.35 (0.13–0.93), indicating how much caregiving Japanese women exert for their parents’ health, in expense of their own health [33]. Despite the lower prevalence of cigarette smoking and heavy drinking among women who were living with parents, those women reported more care-giving worries and were more likely to adopt a sedentary behavior and not receiving regular health check-ups [7]. Women living with parents or in 3-generation households (with parents, spouse, and children) showed a higher risk of coronary heart disease when compared with women living with spouses only; the multivariable HR was 4.94 (1.81–13.5) and 200 (1.01–3.94), respectively [5].

Moreover, there was an absence of an increased risk of stroke in men who lost a spouse, in cases where they also gained other categories of family members. Contrary, the risk of stroke in women who lost their spouses increased further when this loss was accompanied by the gain of parents. Living with other relatives after losing a spouse may cushion the psychosocial stress to some extent in men, but may exacerbate it in women. A recent study showed that living with parent(s) after marital transition exacerbated the stroke risk among women [18]. Although we should expect women to receive similar buffering mechanisms that may cushion their psychosocial stress, like in men, however, this is not the case for the Japanese society. Women who have lost their spouses and shifted to live with parents usually have a high risk of stroke [5–7, 15]. Again, the gender role norms in Japan (male breadwinner and female caretaker and emotional supporter) [31, 32] might compensate men who lost their spouse if they are emotionally supported by parent(s) [34]. However, after losing the breadwinner in their household, women who lost their spouse are at risk to lose their financial stability that is unlikely to be compensated by their elderly parent(s), and for most cases, the opposite is true, those women might be obligated to support their parent(s) financially [18, 31].

We observed an increased risk in women who have gained children while losing other family members; mainly a spouse. It was reported that the risk of stroke has been exacerbated when women turned to live with their children after losing their spouse [6,7,18]; which reflects the lack of financial security. In addition, Tekeda et al, showed that women living with child(ren), even in nuclear families, have a higher score of ‘care-giving and human relations worry’ than those living with their spouses alone [7].

This study is first to report the association of changes in living arrangement, in its three directions; losing, gaining and mixed losing and gaining, with risk of stroke and its types in a dynamic community like in Japan. Limitations of this study include the lack of assessment of potential confounders, such as socioeconomic status (i.e. education and income levels), personality and negative emotions. In addition, the overlap in the categorization of participants in the subgroups analysis may have confounded the results. However, several measures were taken to control for overlap, such as adjusting for the loss or gain of any other categories of family members other than the one targeted in a given category, and the sensitivity analyses among participants who experienced only one change (no overlap) yielded similar results. Also, the lack of incidence data in metropolitan cities, with more nuclear families where family connections are not as tight as in rural areas may reduce the generalizability of the findings. Finally, health impacts may differ by whether the changes in living arrangement were due to pleasant (marriage, remarriage, having a child, travel for work or study, return of travelling member, and etc.) or unpleasant causes (separation, divorce, death, shifting in to take care of a sick family member, and etc.); however, we could not differentiate these situations owing to a lack of information about the reason for changes in the living arrangements.

In conclusion, compared with those living in a stable family household, men who lost a spouse, women who gained parent(s), and women who experienced the combination of losing a spouse while gaining new family member(s), mainly child(ren) or parent(s) were at higher risks of developing stroke.

## Supporting information

S1 Table Adjusted Hazard Ratios for Incident Stroke in Japanese Women Who Have Lost a Spouse While Gaining Children and/or Parents (DOC)
