# Supplementary material for: Changes in the living arrangement and risk of stroke in Japan; does it matter who lives in the household? Who among the family matters?
Source: PLoS One. 2017 Apr 13;12(4):e0173860. doi: 10.1371/journal.pone.0173860 (PMC5390969; doi:10.1371/journal.pone.0173860)
Supplement: S1 Table — (DOC) [file pone.0173860.s001.doc]

**S1Table. Adjusted Hazard Ratios for Incident Stroke in Japanese Women Who Have Lost a Spouse While Gaining Children and/or Parents**

|  | Changes in Living Arrangement Among Women* | | |
| --- | --- | --- | --- |
|  | No Change | Losing a Spouse Gaining a Child(ren) | Losing a Spouse Gaining a Parent(s) |
| Women at risk, *n* | 21722 | 374 | 236 |
| Pearson-year | 301153 | 5081 | 3296 |
| Total Stroke |  |  |  |
| Cases, *n* | 829 | 29 | 12 |
| Model 1 ^a^ | 1.00 | 1.58 (1.10-2.30) | 1.44 (1.03-3.19) |
| Model 2 ^b^ | 1.00 | 1.57 (1.08-2.27) | 1.39 (1.02-3.08) |
| Model 3 ^c^ | 1.00 | 1.57 (1.08-2.28) | 1.39 (1.02-3.12) |
| Hemorrhagic Stroke |  |  |  |
| Cases, *n* | 829 | 15 | 7 |
| Model 1 ^a^ | 1.00 | 2.06 (1.22-3.47) | 1.64 (1.04-3.48) |
| Model 2 ^b^ | 1.00 | 2.01 (1.20-3.25) | 1.53 (1.00-3.25) |
| Model 3 ^c^ | 1.00 | 1.98 (1.17-3.34) | 1.55 (0.99-3.39) |
| Ischemic Stroke |  |  |  |
| Cases, *n* | 829 | 14 | 5 |
| Model 1 ^a^ | 1.00 | 1.24 (0.73-2.12) | 0.91 (0.38-2.21) |
| Model 2 ^b^ | 1.00 | 1.27 (0.75-2.17) | 0.91 (0.38-2.21) |
| Model 3 ^c^ | 1.00 | 1.29 (0.76-2.21) | 0.90 (0.37-2.19) |

^a^ Model 1 Adjusted for age and residential area.

^b^ Model 2 Adjusted further for histories of hypertension, diabetes and use of cholesterol-lowering drugs, number of cohabitants at baseline and job status.

^c^ Model 3 Adjusted further for body mass index, physical activity, smoking status, ethanol intake, perceived psychological stress, life enjoyment and number of cohabitants at baseline time.

*Women at risk of losing a spouse while gaining both a child(ren) and a parent(s) numbered only 23, and no stroke case was observed among them.
